# Validierung subjektiver Beschwerden: Differenzialdiagnostik von „gesundem Leiden“ am Beispiel Müdigkeit

**DOI:** 10.1007/s00103-024-03963-w

**Published:** 2024-10-14

**Authors:** Michael Linden

**Affiliations:** Medizinische Klinik m. S. Psychosomatik, CBF, FPR, Hs. IIIA, Charité Universitätsmedizin Berlin, Hindenburgdamm 30, 12200 Berlin, Deutschland

**Keywords:** Erschöpfung, Biorhythmus, Lebensstil, Differenzialdiagnose, Diagnostische Kriterien, Exhaustion, Biorhythm, Lifestyle, Differential diagnosis, Diagnostic criteria

## Abstract

Subjektive Klagen haben in der Medizin eine große Bedeutung bei der Diagnose und Schwerebeurteilung von Krankheiten, wie auch mit Blick auf die Therapiewahl und -steuerung. Es handelt sich um „Beschwerden“, die vom Patienten berichtet werden, für die es aber keine Objektivierungsmöglichkeit gibt. Sie unterliegen vielfältigen Verfälschungsmöglichkeiten und erfordern daher eine Beschwerdenpräzisierung und Beschwerdevalidierung. Beispiele sind Klagen über Schmerzen, Herzinsuffizienz, schlechte Stimmung oder Müdigkeit. Zudem ist zwischen „krankem“ und „gesundem Leiden“ zu unterscheiden, um eine Medikalisierung von Alltäglichkeiten und diagnostische und therapeutische Fehlschlüsse zu vermeiden. Es werden diagnostische Kriterien für „gesundes Leiden“ beschrieben, da diese Diagnostik eine wichtige Aufgabe in der Medizin ist. Dies wird in diesem Übersichtsartikel am Beispiel von Müdigkeitsklagen ausgeführt.

## Abstract (en)

Subjective complaints are of great importance in medicine. They have an impact on the diagnosis of illnesses, the assessment of illness severity, and treatment selection. There are no objective criteria for such symptoms, and clinicians have to rely on the report of patients only. Examples are complaints about pain, cardiac insufficiency, mood, or fatigue. There are many possibilities for distorted reports. Clinicians must therefore clarify what is meant and validate patient reports. A discrimination must be made between signs of illness and “healthy suffering,” to avoid medicalization of daily nuisances and to avoid misdiagnoses and inadequate treatments. Diagnostic algorithms for healthy suffering are described, as this diagnosis is of importance in medicine. This is outlined using the example of fatigue.

Zum menschlichen Alltag gehören Missempfindungen und Bagatellbeschwerden jeder Art, von schweren Beinen über Hautveränderungen, Verdauungsprobleme, Schmerzen, Erschöpfung, Kraftlosigkeit bis hin zu Lustlosigkeit, schlechter Stimmung, Ängsten und vielem mehr. Nimmt man die bevölkerungsbezogenen Normdaten der Symptomcheckliste 90 [1] als Referenz, dann geben die Befragten im Durchschnitt an, unter etwa 20 der 90 aufgelisteten Symptome zu leiden. Sie können bedeutungslos sein, aber auch auf schwerwiegende Krankheiten hindeuten. Ein Beispiel für eine derartige Alltagsklage ist Müdigkeit. Es handelt sich um ein subjektives Erleben, das komplexe Fragen zur Diagnostik sowie den therapeutischen Konsequenzen aufwirft. In diesem Beitrag soll zunächst allgemein die Bedeutung subjektiver Klagen und Beschwerden dargestellt werden, dann die differenzialdiagnostischen Probleme am Beispiel von Müdigkeitsklagen, das Problem von Verfälschungsmöglichkeiten subjektiver Klagen, die Notwendigkeit und Methoden einer Beschwerdenvalidierung und schließlich Kriterien für gesundes Leiden, d. h. auch gesunde Müdigkeit.

## Subjektive Beschwerden und Klagen

Subjektive Klagen haben in der Medizin eine große Bedeutung bei der Diagnostik, der Schwerebeurteilung von Krankheiten wie auch der Therapiesteuerung [2, 3]. Dies sind Beschwerden, die ausschließlich auf dem Bericht der Patienten basieren und für die es keine Objektivierungsmöglichkeit gibt. Dabei gilt es ärztlicherseits herauszufinden, welche Problematik sich hinter der Klage verbirgt und wie sie diagnostisch zu werten ist. Auch stellt sich die Frage, ob die Beschwerden tatsächlich vorliegen oder nur geklagt werden.

Ein Beispiel ist die Klage über Müdigkeit. Das Problem beginnt mit der semantischen Konnotation des Begriffs und der unterschiedlichen Verwendung durch Patienten und Fachleute. Laut der Internetplattform „Woxikon“ ergibt die Suche nach Synonymen für „Müdigkeit“ 245 Begriffe in 18 Symptomgruppen. Beispiele sind Schwächegefühl, Erschöpfung, Schlappheit, Antriebsmangel, Energiemangel, Schläfrigkeit, Schlafbedürfnis, Ruhebedürfnis, gedrückte Stimmung, Konzentrationsprobleme, reduzierte geistige Aktivität, muskuläre Schwäche, Abgeschlagenheit, Zerschlagenheit, Mattigkeit, Abgespanntheit u. v. m. Hinzu kommen Fachbegriffe, die ihren Weg zum Teil auch in die Alltagssprache gefunden haben und dort oft mit idiosynkratischem Verständnis verwendet werden, wie z. B. Burn-out, Chronic Fatigue, myalgische Enzephalomyelitis usw.

Wie schwer eine Begriffsklärung ist, zeigt beispielsweise die „S3-Leitlinie Müdigkeit“ (Register-Nr. 053-002) der Arbeitsgemeinschaft der Wissenschaftlichen Medizinischen Fachgesellschaften (AWMF), in der einerseits durchgehend von Müdigkeit gesprochen wird, andererseits jedoch an keiner Stelle eine klare Definition gegeben wird, sondern stets nur auf Zusatzprobleme Bezug genommen wird. Es wird im Abschnitt 4 als Definition nur gesagt, dass Müdigkeit eine universelle menschliche Erfahrung ist, die in verschiedenen Formulierungen wie Schlappheit, Mangel an Energie, Erschöpfung (Fatigue), Ermüdung, frühe Ermüdbarkeit, (Tages‑)Schläfrigkeit, Einschlafneigung tagsüber usw. vorgetragen wird. Ähnliche semantische Probleme stellen sich auch bei anderen subjektiven Klagen, wie z. B. Schmerz, depressiver Verstimmung oder Appetitlosigkeit, und sind ein Grundproblem der Medizin. Eine präzise Begriffsklärung erscheint im konkreten Fall damit unerlässlich.

Auch wenn eindeutig ist, was der Klagende meint, ist die klinische Wertigkeit zunächst einmal weiterhin offen. So werden beispielsweise nach der Klassifikation der „New York Heart Association“ auf der Basis des subjektiven Belastungserlebens, d. h. Klagen über Erschöpfung und Leistungsunfähigkeit, 4 Schwerestufen der Herzinsuffizienz unterschieden [4–6]. Herzinsuffizienz ist im klinischen Alltag somit im Wesentlichen eine „psychosomatische“ Erkrankung, die vorrangig auf subjektivem Beeinträchtigungserleben basiert, das weitgehend unabhängig von objektiven Parametern ist, wie dem Herzauswurfvolumen. Es ist im Einzelfall daher zunächst einmal offen, ob die subjektive Klage somatische Schwerestufen der Herzerkrankung abbildet oder ob es um ein davon unabhängiges psychisches Erleben geht oder im Begutachtungskontext sogar um Präsentationsklagen. d. h. Beschwerden, die jemand anderem gegenüber (z. B. dem Gutachter) präsentiert werden. Dieses Beispiel zeigt die Bedeutung subjektiver Klagen in der Medizin generell und dies selbst bei gesicherten somatischen Erkrankungen.

Auch standardisierte Instrumente können das Problem der Subjektivität nicht lösen. Dies gilt zum einen für Selbstbeurteilungsfragebögen, die ihrer Natur nach auch nur das Patientenerleben erfassen. Aber auch Fremdbeurteilungen und standardisierte Interviews basieren zu einem wesentlichen Teil auf Patientenklagen und nur zu einem geringen Teil auf Fremdbeobachtungen der Untersucher im engeren Sinne [7]. Standardisierte Instrumente können sogar noch zu Zusatzproblemen führen, da sie neben der eigentlich interessierenden Leitklage immer auch eine Fülle sonstiger Beschwerden auflisten, womit die Spezifität infrage zu stellen ist. So wird beispielsweise in der „Fatigue-Skala“ [8, 9] gefragt: Ist Müdigkeit ein Problem für Sie? Müssen Sie öfter ausruhen? Fühlen Sie sich müde oder schläfrig? Haben Sie Schwierigkeiten, Dinge in Angriff zu nehmen? Mangelt es Ihnen an Energie? Haben Sie weniger Kraft in Ihren Muskeln? Fühlen Sie sich schwach? Fällt es Ihnen schwer, sich zu konzentrieren? Passieren Ihnen Versprecher beim Reden? Fällt es Ihnen schwer, klar zu denken? Haben Sie Gedächtnisprobleme? Dies sind subjektive Beschwerden allgemeiner Art, die neben Müdigkeit auch Ausdruck vieler anderer mentaler und somatischer Zustände sein können.

Auch in dem Patientenfragebogen der S3-Leitlinie „Müdigkeit“ der AWMF erscheint der Begriff Müdigkeit nur in der Überschrift. Gefragt wird dann nach dem Gefühl, gesund zu sein, der Kontextabhängigkeit des eigenen Befindens, körperlicher Bewegung, Belastungsintoleranz, Gewicht und Appetit sowie Stuhlgang. Auch alle diese Angaben sind subjektiver Natur, entziehen sich einer Objektivierung, können müdigkeitsassoziiert sein oder auch nicht (z. B. Mangel an Proaktivität, Stuhlgang). Es wird ein Syndrom abgefragt, dass eher allgemein ist, und damit das Spektrum der differenzialdiagnostischen Denkmöglichkeiten eher noch erweitert, als dass dadurch zu diagnostischer Klarheit beigetragen wird. Was führt Menschen dazu, solche Fragen zu bejahen? Welche Ursache oder Krankheit verbirgt sich dahinter oder sind die Befragten gesund und „einfach nur müde“, wie dies beispielsweise bei Müttern oder Vätern mit Kleinkindern nicht unüblich ist?

Will man „Müdigkeit“ als singuläres Symptom beschreiben, dann handelt es sich um das Bedürfnis, die Augen zu schließen und sich zur Ruhe zu begeben. Im Psychopathologieglossar der Arbeitsgemeinschaft für Methodik und Dokumentation in der Psychiatrie [10] wird Müdigkeit den Vitalstörungen zugeordnet und explizit abgegrenzt von sonstigen Symptomen, die oft in einem Atemzug mit Müdigkeit genannt werden, wie z. B. Konzentrationsstörungen oder Antriebsstörung bzw. der Schwierigkeit, Aktivitäten zu beginnen und aufrechtzuerhalten. Zur Erläuterung mag das Beispiel eines Arztes im Nachtdienst dienen, der müde sein und dennoch hoch konzentriert und sehr aktiv sein kann, im Vergleich zu einem lustlosen Arzt, der antriebslos und unkonzentriert sein kann, ohne sich müde zu fühlen.

## Differenzialdiagnostik von subjektiven Klagen am Beispiel von Müdigkeit

Müdigkeit ist ein geradezu ubiquitäres Symptom [11]. In der ICD-11 (Internatinal Classification of Diseases, 11. Rev) [12] gibt es das Kapitel 21 (M-Codes) für unspezifische und uneindeutige Beschwerden unter der Überschrift „Allgemeine Symptome (General Symptoms)“. Darunter fallen subjektive, medizinisch nicht eindeutig zuzuordnende somatische und psychische Symptome, aber auch unklare Laborwerte. Des Weiteren gibt es das Kapitel 24 (Q-Codes) für „Faktoren, die den Gesundheitsstatus beeinflussen und auch zur Inanspruchnahme des Gesundheitswesens führen“, die aber keine „Krankheit, Verletzung o. ä.“ sind.

Hierunter findet sich der Code „MG22“ für „Müdigkeit (Fatigue)“. Es wird dort kein Symptom, sondern ein Syndrom beschrieben mit dem Gefühl der Erschöpfung, Lethargie oder reduzierten Energie, typischerweise begleitet von dem Gefühl eingeschränkter oder reduzierter somatischer oder mentaler Ressourcen, einhergehend mit einer eingeschränkten Arbeitsfähigkeit und reduzierten Fähigkeit, auf Herausforderungen zu reagieren. Es wird gesagt, dass Müdigkeit normalerweise auf somatische oder mentale Anstrengung folgt, aber auch unabhängig davon auftreten kann i. S. eines unspezifischen Befindlichkeitszustands. Es wird dann noch eine Reihe von Ausschlussformen genannt wie Chronic-Fatigue-Syndrom, myalgische Enzephalitis, postvirale Erschöpfung, Müdigkeit nach Kampferfahrungen, Müdigkeit bei Hitze, Depression, Schlafstörungen, Senilität, Schwangerschaft, Anstrengung, somatische Überforderung. Diese Ausschlusstatbestände umfassen jedoch nicht das gesamte Spektrum der sekundären Fatigue-Syndrome.

Im ICD-11-Browser wird das Stichwort „Fatigue (Müdigkeit)“ noch weitere 38-mal genannt [12]. Müdigkeit wird darüber hinaus auch noch unter sonstigen Bezeichnungen abgebildet. Ein Beispiel von besonderer Relevanz sind Klagen über Schlafstörungen, die ihrer Natur nach auch mit Müdigkeit assoziiert sind [13]. Es werden etwa 200 verschiedene Schlafstörungen unterschieden [14] mit Prävalenzraten von etwa 10–50 % in der Bevölkerung sowie Beeinträchtigungen am Tage bei etwa 10–15 % [15–17].

Diese verschiedenartigen Nennungen von Müdigkeit spiegeln das klinische Alltagswissen wider, dass Müdigkeit ein Begleitsymptom vieler körperlicher und psychischer Erkrankungen sein kann. In der S3-Leitlinie „Müdigkeit“ der AWMF findet sich dazu eine umfangreiche Auflistung. Danach klagen etwa 2 Drittel der Patienten mit depressiven Störungen, somatoformen Störungen, Malignomen, Lebererkrankungen oder nach viralen Infektionen auch über Müdigkeit [18]. Müdigkeit ist aber auch in der Allgemeinbevölkerung ein häufiges Phänomen. Etwa ein Drittel bis die Hälfte aller Menschen klagen über Fatigue/Müdigkeit und diese Beschwerde ist auch einer der häufigen Beratungsanlässe im hausärztlichen Bereich [19].

Diese epidemiologischen Daten zur Prävalenz von Müdigkeitsklagen deuten darauf hin, dass nicht zwingend in jedem Fall von einem Krankheitszustand ausgegangen werden darf. Differenzialdiagnostisch stellt sich die Frage, in wie vielen Fällen von „gesunder Müdigkeit“ [20] auszugehen ist. Müdigkeit begleitet Menschen natürlicherweise über den ganzen Tag. Dies beginnt mit dem Aufstehen und der Hoffnung, dass eine analeptische Selbstmedikation mit Coffein oder Thein die Müdigkeit überwinden hilft, gefolgt vom Schlaf in der Bahn auf dem Weg zur Arbeit, dem Mittagsschlaf und dem erneuten Wunsch nach einem Kaffee, der Abendmüdigkeit und schließlich der Bettschwere. Müdigkeit ist somit „normal“. Grundlage ist ein Biorhythmus, der im Hypothalamus und Nucleus suprachiasmaticus hormonell reguliert wird [21, 22]. Neben dem Tagesrhythmus gibt es bei etwa 70 % der Menschen auch noch einen saisonalen Biorhythmus mit jahreszeitlichen Aktivitätsschwankungen, analog zum Winterschlaf mancher Tiere [23, 24].

Der endogene Müdigkeitsbiorhythmus wird zusätzlich durch die Umwelt beeinflusst, wie die Jetlag-Adaptationen zeigen [25, 26]. Schlafstörungen und Müdigkeit können daher auch verhaltensabhängig sein, etwa wenn jemand zu spät ins Bett geht. Von Bedeutung sind auch die allgegenwärtige künstliche Beleuchtung, Blaulicht-Abstrahlungen von Bildschirmen oder das Aufwachen nach Dienstplan und nicht in Abhängigkeit davon, ob man ausgeschlafen hat [27].

Ein anderes Beispiel für ein unspezifisches nicht krankhaftes Müdigkeitserleben ist Burn-out (ICD-11: QD85), der im ICD-11 charakterisiert wird als ein Gefühl des Energieverlusts und der Erschöpfung, was von Patienten auch als Müdigkeit beklagt werden kann. Zugleich ist es ein Syndrom, das in der ICD-11 explizit als Ausdruck einer negativen und zynischen Haltung gegenüber dem eigenen Beruf und Unzufriedenheit mit der beruflichen Leistung beschrieben wird, was mit Müdigkeit im eigentlich Sinne nicht unbedingt etwas zu tun hat. Solche Beispiele zeigen, dass sich bei subjektiven Klagen, wie z. B. über Müdigkeit, immer auch die Frage stellt, was das eigentliche Problem ist und welche Motive der Klage zugrunde liegen.

## Verfälschung subjektiver Klagen

Bei der Beurteilung subjektiver Klagen und Symptomschilderungen ist differenzialdiagnostisch immer zu berücksichtigen, dass der Beschwerdevortrag auf vielfältige Weise verzerrt sein kann [28, 29]. Es gibt die *Simulation*, d. h. die bewusste Vortäuschung von nicht existierenden Beschwerden, die mit Absicht vorgetragen werden, zumeist um ein bestimmtes Ziel zu erreichen, beispielsweise zur Erlangung eines Arbeitsunfähigkeitsattestes. In der ICD-11 gibt es dafür den Code QC-30 (Malingering), in der ICD-10 den Code Z76.5. In gutachterlichen Kontexten ist damit in etwa jedem zweiten Fall zu rechnen [30]. Davon abzugrenzen sind *artifizielle Störungen* (ICD-10 68.1), d. h. die Präsentation oder sogar Erzeugung von Beschwerden, um einen Krankheitszustand herzustellen, meist als Ausdruck und Lösungsversuch für unbewusste Konflikte [31]. Somatoforme Störungen oder „medizinisch unerklärte somatische Symptome“ können Ausdruck einer *Somatisierungsstörung* sein, d. h. einer Angsterkrankung i. S. einer erhöhten Angst um die körperliche Unversehrtheit, oder einer *somatoform autonomen Störung* i. S. einer konstitutionell erhöhten vegetativen Labilität.

Die Beschwerdenschilderung kann einer *Verdeutlichungstendenz* unterliegen [32]. Die Betroffenen versuchen in der Interaktion mit anderen Menschen, seien es Ärzte, Lebenspartner oder Kollegen, Verständnis für ihre Situation dadurch zu erlangen, dass sie subjektives Leiden eindrücklicher, d. h. deutlicher schildern. Die Verdeutlichungstendenz ist alltäglich im Umgang mit Patienten und verwandt mit der *Aggravation*, d. h. einem aufbauschenden Beschwerdenvortrag. Von Relevanz ist auch die *emphatische Beschwerdenschilderung*, d. h. der verdeutlichende Hinweis auf eigenes Leiden als Form eines Vorwurfs gegen andere und damit Ausdruck der eigenen Opferrolle [33]. Derartige subjektive Klagen dienen aber auch für den Betroffenen selbst als Beleg, dass ein äußeres Ereignis „wirklich schlimm“ war (z. B. wäre man nicht müde, könnte man nicht darüber klagen, dass man viel arbeiten muss, weil es dann ja nicht schlimm wäre).

Beschwerdenvorträge können auch einer *Tendenz zur sozialen Erwünschtheit* unterliegen. Wenn ein Therapeut bestimmte Symptome besonders ernst nimmt, wird auch der Patient diese für wichtig erachten und hervorheben. In Abhängigkeit von der individuellen Weltanschauung gibt es auch eine *kulturabhängige Symptompräsentation*. Menschen mit eher somatischem Verständnis werden ihr Leid mit somatischen Beschwerden zum Ausdruck bringen; Menschen mit psychosozialen Konzepten werden psychisch klagen. Dies ist u. a. in der transkulturellen Medizin von großer Bedeutung.

Ein wichtiger psychologischer Prozess ist die Fixierung der eigenen Aufmerksamkeit auf die eigene Befindlichkeit im Sinne der *Lage-Handlungs-Orientierung* [34, 35]. Lageorientierte Menschen haben eine Tendenz, ihre Aufmerksamkeit auf die eigene Befindlichkeit zu konzentrieren, was auch zu einer verstärkten Wahrnehmung von somatosensorischen und psychischen Alltagsbefindlichkeiten führt. Damit verwandt ist die subjektive Verarbeitung von Allgemeinbeschwerden, was beispielsweise unter dem Begriff der *Willensanstrengung* in der Sozialmedizin umfangreich diskutiert wird [36]. So kann beispielsweise Müdigkeit „überspielt“ werden oder auch nicht, abhängig von der Motivationslage [37].

Dabei sind *Rahmenbedingungen* von Bedeutung. Müdigkeit, Erschöpfungs- und Überforderungsgefühle können alleine schon beim Gedanken daran entstehen, dass bei der Arbeit Schwierigkeiten, Konflikte oder ungeliebte Anforderungen warten und dass die Arbeit weniger als Lust, denn als Belastung erlebt wird. Der Begriff „Burn-out“ (ICD-11: QD85) impliziert, wie oben bereits beschrieben, eine solche arbeitsbezogene Lustlosigkeit [38].

Schließlich ist von Bedeutung, dass Unerklärtes zwingend Angst auslöst, d. h. psychologisch gesprochen ein *„unbedingt angstauslösender Stimulus“* ist [39]. Über diesen psychologischen Mechanismus können uneindeutige und „unerklärte“ Beschwerden Angst auslösen. Wenn dann ärztliche diagnostische und therapeutische Leistungen in Anspruch genommen werden mit der Gefahr, dass „etwas gefunden wird“, oder Allgemeinsymptome nun medizinisch schwerwiegend gedeutet werden, dann kann es mit therapeutischer Unterstützung zu einer Beschwerdenaggravation kommen i. S. einer „Nocebo-Reaktion“ [40, 41]. Ein Beispiel ist die Diskussion um Chronic Fatigue, Fibromyalgie, myalgische Enzephalomyelitis oder das Post-Covid-Syndrom. Manche Konzepte können Patienten durch komplizierte Vermutungen zu Krankheitsprozessen mit weitreichenden und einschränkenden Verhaltensempfehlungen erheblich ängstigen, z. B. das Konzept des „Batterie-Crashs“ bei Post-Covid, mit der ängstigenden und patientenschädigenden Behauptung, dass es bei Bewegung zu schlimmsten Folgen käme, obwohl es im Körper keine Batterie gibt und Schonhaltungen krankheitsfördernd sind [42–44]. So können auch Bagatellbeschwerden *medikalisiert* werden [30, 45] mit der Folge von Labeling [46] der Betroffenen, Fehlbehandlungen, Unterminierung der Selbstwirksamkeit oder negativen sozialen Konsequenzen.

Beschwerden von Patienten und ihre Wahrnehmung durch Therapeuten sind schließlich auch modeabhängig. So finden sich in Zeitschriften regelmäßig Beiträge zur Gefährlichkeit von Stress oder Burn-out und zur erschöpften, überforderten und gestressten deutschen Nation (Stern, z. B. 30.10.2023, 18.12.2023, 27.12.2023). Diese Weltsicht lässt sich zurückverfolgen bis zur Prägung des Begriffs „Neurasthenie“ durch den New Yorker Arzt Beard [47], der darunter eine „nervöse Erschöpfung“ infolge des zunehmenden Straßenlärms und der Alltagshektik oder auch eine Stressreaktion auf Zeitunglesen bei Frauen verstand [47]. Bis vor einigen Jahren war Neurasthenie die häufigste Diagnose in China als Ausdruck von subjektivem Leiden infolge eines engagierten Einsatzes für die Nation [48]. In diesem Sinne ist auch Burn-out eine geradezu ehrenhafte Störung, weil sie zeigt, dass sich der Betroffene engagiert hat [49–51].

Ergänzend ist anzumerken, dass es Beschwerdeverfälschungen nicht nur als Beschwerdenaggravierung gibt, sondern auch in Form von Dissimulation oder geschönter Selbstdarstellung. Dabei sind dieselben psychologischen Mechanismen wirksam wie für die Beschwerdenausgestaltung.

## Beschwerdenvalidierung

Aus den vielfältigen Möglichkeiten der Modifikation von subjektiven Beschwerden ergibt sich, dass Therapeuten derartige Klagen ernst nehmen müssen, sie jedoch nicht unhinterfragt als Tatsachenbeschreibungen hinnehmen dürfen. Es bedarf aus diagnostischen und therapeutischen Gründen, zum Patientenschutz und insbesondere auch in gutachterlichen Kontexten einer „Beschwerdenvalidierung“ [28, 29, 52]. Dabei geht es nicht darum, den Klagenden der „Lüge“ zu überführen. Patientenklagen sind Ausdruck der subjektiven Befindlichkeit und Wahrnehmung der Betroffenen und als solche anzuerkennen und ernst zu nehmen. Dennoch ist die medizinische Wertigkeit der subjektiven Klagen sachlich abzuklären, um Fehldiagnosen und Fehlbehandlungen zu vermeiden.

Ein sozialrechtlich wichtiger Aspekt ist, dass Betroffene selbst niemals sagen können, dass sie krank sind, d. h. unter einer Krankheit im sozialrechtlichen Sinne leiden. Dies kann nur ein „approbierter Therapeut“ entscheiden, d. h. jemand, der die staatliche Befugnis zur Ausübung der Heilkunde hat. Betroffene können nur zum Ausdruck bringen, dass es ihnen nicht gut geht. Mit der Feststellung einer Krankheit wird soziologisch wie rechtlich aus einem leidenden Menschen ein Patient, was im Wesentlichen bedeutet, dass die Leistungspflicht der Sozialversicherungen in Kraft tritt. Wer sich schlecht fühlt im Kontext einer Eheauseinandersetzung, muss psychologische Hilfe selbst zahlen. Wenn der approbierte Therapeut dies als Ausdruck einer depressiven Erkrankung beurteilt, dann zahlt die Krankenkasse. Die Feststellung, dass eine Krankheit vorliegt, ist somit eine gutachterliche Aussage mit weitreichenden therapeutischen und sozialmedizinischen Konsequenzen, weshalb einer Beschwerdenvalidierung eine große Bedeutung zukommt.

Zu diesem Thema gibt es eine Reihe von Gerichtsurteilen, die besagen, dass gutachterliche Aussagen, d. h. auch die Feststellung einer Krankheit, die Prüfung von Verfälschungstendenzen nach den in dem jeweiligen medizinischen Fachgebiet anerkannten Methoden verlangt. Keinesfalls darf sich die Beurteilung eines Krankheitszustandes unkritisch allein auf die Beschwerdeschilderung des Betroffenen stützen (LSG NRW, 8. SenatI.8.R45/17; LSG Niedersachsen/Bremen, I.10.B.62/02; Bayer. LSG vom 11.09.2003, Az.: L 14 RA 103/02; LSG Baden-Württemberg vom 12.12.2002, Az.: L10 RA 4885/00). Die fachliche Beurteilung von subjektiven Beschwerden und die Begründung von Diagnosen muss den allgemeinen Anforderungen an gutachterliche Stellungnahmen entsprechen. Dies sind Plausibilität und Schlüssigkeit, d. h., die Argumentationskette sollte logisch, widerspruchsfrei und aus den vorhandenen Befunden ableitbar sein. Die Beurteilung muss nachvollziehbar sein, d. h. auch von anderen Fachleuten und ggf. sonstigen Interessenten verstanden werden können, wie z. B. Mitarbeitern von Kranken- und Rentenversicherungen oder Richtern und auch vom Patienten selbst.

Indikatoren für Beschwerdenverfälschungen sind, (a) wenn geklagte Beschwerden nach Art und Lokalisation mit den objektiven Befunden nicht in Einklang zu bringen sind, (b) wenn es Diskrepanzen gibt zwischen subjektiver Beschwerdenschilderung (einschließlich Selbsteinschätzung im Fragebogen) und Beeinträchtigung in der Untersuchungssituation, (c) wenn ein geringer Leidensdruck trotz intensiv geschilderter Beschwerden („belle indifference“) besteht, (d) wenn die Schilderung der Beschwerden und des Krankheitsverlaufes vage, wechselhaft und unpräzise ist, (e) wenn Klagen appellativ-demonstrativ vorgetragen werden, ohne dass beim Gutachter das Gefühl des Betroffenseins entsteht, (f) wenn es Diskrepanzen gibt zwischen den vorgetragenen Angaben und fremdanamnestischen Informationen (einschließlich Aktenlage), (g) wenn andauernde Beschwerden geschildert werden, die sich zu keiner Tageszeit bessern, (h) wenn Diskrepanzen zwischen geschilderten Beeinträchtigungen und Aktivitäten des täglichen Lebens (z. B. Hobbys, Urlaube, Fahrtüchtigkeit) bestehen, (i) wenn keine Strategien zur Beschwerdenbewältigung geschildert werden.

In der Fachliteratur werden auch standardisierte und normierte Validierungsmethoden diskutiert [50]. Empfohlen wird beispielsweise, mehrere Instrumente parallel einzusetzen, um einen Vergleich von Klagen auf mehreren Ebenen zu ermöglichen [7]. Es gibt auch spezielle Testverfahren zur Identifizierung von Beschwerdenverzerrungen, wie z. B. der „Strukturierte Fragebogen Simulierter Symptome“ (SIMS; [53]) oder als Fremdbeurteilungsinstrument das „Structured Interview of Reported Symptoms“ (SIRS‑2; [54]). Neben einer größeren Anzahl von tatsächlich möglichen Beschwerden werden darin seltene, aber auch unrealistische Symptome oder Symptomkombinationen wiederholt abgefragt. Von entscheidender Bedeutung ist die klinische Beobachtung über den Verlauf einer Untersuchung, da Klagen vor allem beim Erstkontakt betont werden, im Weiteren dann aber oft „abflauen“ [55]. Auch detaillierte anamnestische Erhebungen, z. B. zu Schlafanamnese, Aktivitäten in Haushalt und Garten, Hobbys und Vereinsleben, Beschäftigung mit Haustieren, sonstige familiäre, soziale, sportliche und sexuelle Aktivitäten, Besuche und Urlaubsreisen, Partnerverhalten, Fähigkeit zum Auto- und Radfahren können wichtige Informationen liefern. Die Intensität und der Verlauf von Beschwerden können auch über Selbstbeobachtungen konkretisiert werden, beispielsweise in Form von Tagebüchern. Schließlich können auch Fremdanamnesen ergänzende Hinweise für die Beschwerdevalidität liefern.

## Differenzialdiagnostik eines „gesunden Leidens“ am Beispiel Müdigkeit

Wie dargelegt, ist die Unterscheidung zwischen Krankheit und gesunden Leidenszuständen von grundsätzlicher Bedeutung in der Medizin. Wenn beispielsweise ein Dermatologe einen Hautfleck als gesunde Hautverfärbung erkennt und von einem Malignom abgrenzt, dann ist das eine wichtige ärztliche Leistung. Auch im § 1 (5) der „Psychotherapie-Richtlinie“ [56] wird explizit gesagt, dass Probleme in der beruflichen Anpassung, Erziehungsprobleme, Paar- und Familienprobleme, sexuelle Probleme, körperliches Unwohlsein oder allgemeine Behinderungen im Leben keine Krankheiten und damit auch kein Gegenstand für Psychotherapie sind. Subjektive Beschwerden können Krankheitszeichen sein, sie können aber auch „gesundes Leiden“ sein [57–59]. Es gehört zum alltäglichen menschlichen Leben sich „schlecht“ zu fühlen, beispielsweise nach anstrengender Arbeit, bei Auseinandersetzungen mit anderen Personen, nach einem Todesfall, bei einem finanziellen Verlust, beim Erleben von Ungerechtigkeit oder Herabwürdigung, bei Enttäuschungen u. v. m. Die Abgrenzung zwischen gesund und krank, beispielsweise krankhafter und gesunder Müdigkeit, basiert zum einen auf der vorbeschriebenen Beschwerdenvalidierung. Zusätzlich sind aber auch positive Feststellungen für die „Diagnose Gesundheit“ erforderlich. Tab. 1 gibt eine Übersicht über diagnostische Kriterien für gesundes Leiden.

1. Im ersten Schritt ist, wie oben bereits beschrieben, die Konnotation der verwendeten Begriffe und Symptombezeichnungen zu klären. Derselbe Begriff kann viele Bedeutungen haben und für denselben Zustand können viele Begriffe zur Verwendung kommen. So ist bei Klagen über „Müdigkeit“ unterschiedlich zu bewerten, ob faktisch eine Herabgestimmtheit, Augenbrennen, Lustlosigkeit oder ein Müdigkeitssymptom im engeren Sinne vorliegen. Es ist also zu explorieren, was der Klagende erlebt, wenn er „Müdigkeit“ sagt. Für gesunde Müdigkeit spricht das Bedürfnis nach Ruhe oder Schlaf, unabhängig von Antriebs- oder Aktivitätsbeeinträchtigungen.
2. Es ist dann zu klären, ob die vorgetragene Klage einem (psycho)pathologischen Symptom im engeren Sinne entspricht. So gibt es etwa 500 Formen schlechter Stimmung, von denen aber nur wenige als pathologisch einzuordnen sind (z. B. Lustlosigkeit, Missmut, Trauer, schlechte Stimmung u. v. m. im Gegensatz zu depressiver Verstimmung, Panikgefühlen, Parathymie).
3. Es ist zu erfassen, welche Zusatzsyndrome geklagt werden. Ergibt sich eine klinisch bekannte Syndromgestalt oder werden multiforme arbiträre Beschwerden vorgetragen?
4. Es ist die Intensität der Beschwerden zu klären. Ein wenig Müdigkeit ist belanglos, schwere Müdigkeit etwas anderes. Die Bedeutung gerade von subjektiven Beschwerden ist sehr unterschiedlich in Abhängigkeit von der Intensität der Beschwerden. Dieses Problem findet in der diagnostischen Literatur nur unzureichend Beachtung, wo Symptome unabhängig von der Intensität gleich bewertet werden [60]. Gesunde Müdigkeit wird als beeinträchtigend erlebt, ist jedoch im Alltag eher nicht behindernd.
5. Ein weiteres Kriterium ist die Leidenstoleranz oder Resilienz [61], d. h. die Widerstandsfähigkeit gegenüber Anforderungen, Misslichkeiten oder Unwohlsein. Ein Mangel an Resilienz und eine erhöhte Tendenz zur Klagsamkeit ist für sich genommen keine Krankheit, sondern ein Persönlichkeitsstil. Ein faktenbasierter Beschwerdevortrag erfolgt in der Regel auf eher nüchterne und nicht auf aggravierende Art.
6. Es ist der Grad der Selbstbeobachtung abzuklären. Es ist eine Persönlichkeitseigenschaft, inwieweit Menschen eher handlungsorientiert sind oder eher eine verstärkte Lageorientierung haben [34, 35]. Letztere nehmen schneller einmal eine warme Stirn oder einen rauen Hals war, was nicht krankhaft ist. Die verstärkte Aufmerksamkeit auf die eigene Befindlichkeit führt zwingend zu einer verstärkten Wahrnehmung der aktuellen Befindlichkeiten, einer erhöhten Sensibilität und auch Selbstfürsorge, ohne dass dies ein krankhafter Prozess ist.
7. Es ist ein normalpsychologischer und nicht krankhafter Prozess, dass Unerklärtes verunsichert, zur verstärkten Selbstbeobachtung und damit auch Verstärkung somatosensorischer Wahrnehmungen führen kann, mit der Folge einer weiteren Beängstigung. Eine Reaktion und eine Bewältigungsmöglichkeit bei Verunsicherung sind beliebige subjektive Erklärungen. Die Betroffenen wissen dann vermeintlich, aber mit hoher subjektiver Überzeugung, was sie haben, woher es kommt und was zu tun ist, z. B. Folge falscher Ernährung, Sick-Building-Syndrom, Elektrosmog, Arbeitsüberlastung, Berufskrankheit u. v. m. [62. 63]. Hierbei ist auch zu klären, wo diese „Erklärungen“ herkommen und welche Rolle ärztliche „Nocebo-Kommunikationen“ dabei spielen. Gesunde Müdigkeit kann auch mit Besorgnis hinsichtlich einer bestimmten Ursache einhergehen, ohne dass dies eine Krankheitswertigkeit begründet.
8. Von Bedeutung sind auch persönlichkeitsnahe Krankheitskonzepte bzw. dysfunktionale Kognitionen. Ein Beispiel sind internale Kontrollüberzeugungen, d. h. die Annahme, dass das eigene Schicksal vom eigenen Tun abhängt, während externale Kontrollüberzeugungen äußere Faktoren für das eigene Befinden verantwortlich machen, seien es das Schicksal oder andere Personen [64]. Internal kontrollierende Menschen können eine geringe Leidenstoleranz haben und aktiv gegen Beschwerden angehen, aber dann mit Ärger bis zur Verzweiflung und Aggravierung reagieren, wenn die Symptome dem eigenen Willen „nicht folgen“ und sich die Beschwerden nicht abstellen lassen. External attribuierende Personen können hingegen bei Unpässlichkeiten darin „baden“ und sich über das unfaire Schicksal beschweren. Daraus resultierender „Ärger“ über Müdigkeit darf nicht mit krankhafter Müdigkeit verwechselt werden.
9. Von besonderer Relevanz ist der Kontext der Beschwerdenentstehung. Im ICD-11 gibt es die M‑ oder Q‑Codes für Beschwerdenanlässe ohne Krankheitswert, wie z. B. Probleme mit Bezug auf das Berufsleben oder Probleme mit Bezug auf die Partnerschaft oder Schwierigkeiten bei der Lebensbewältigung. Es gibt keinen Menschen ohne solche Schwierigkeiten und ohne solches subjektives Leiden. Der Entstehungskontext der Beschwerde ist zu klären. Dies beinhaltet bzgl. Müdigkeit die Tageszeiten und -aktivitäten, Schlaf, Anforderungen, zeitliche Charakteristika, Frequenz u. v. m.
10. Von Bedeutung ist im Weiteren der Umgang mit den geklagten Beschwerden und eventuellen Einschränkungen. Dazu gehören eigene Anstrengungen, aktiv auf die Beschwerden einzuwirken, wie andererseits aber auch ein „giving up“, das Aufgeben jeglicher Gegenwehr [65]. Insbesondere inadäquates Schonverhalten ist zu berücksichtigen. Inaktivität kann zu körperlicher wie mentaler Schwäche führen und dies nicht nur nach Bettlägerigkeit, was dann nur durch ein längerfristiges Training wieder zu bessern ist [66]. Wer mangels körperlicher Aktivität sich beim Treppensteigen erschöpft fühlt, ist nicht krank. Inadäquat kann aber auch eine kämpferische Gegenreaktion sein, z. B. in Form von Ärger über die Befindensstörung und sofortigen Gegenmaßnahmen, wie z. B. Tabletten bei Muskelkater.
11. Zu beurteilen ist die Funktionalität der Klage, was gelegentlich unter dem Begriff des Krankheitsgewinns beschrieben wird [67]. Subjektives Unwohlsein hat in aller Regel auch eine soziale Dimension. Wer den Chef und die Arbeit nicht mag, sich dadurch belastet fühlt und die Arbeit vermeiden möchte, kann dies sanktionsfrei nur mit einer „Krankschreibung“ erreichen, was wiederum einen Klagevortrag erfordert.
12. Es wurde oben bereits auf die Bedeutung gesellschaftlicher oder professioneller Normen und Deutungen von Beschwerden hingewiesen [40, 41]. Homosexualität war eine Krankheit bis sie auf gesellschaftlichen Konsens hin zur Normalität erklärt wurde. Vitiligo war keine Krankheit bis sie unter dem Code L80 in die ICD aufgenommen wurde. Neben solchen offiziellen Verkodierungen spielen auch kulturelle Erwartungen eine Rolle. Der Ausdruck von Leiden und die Bedeutung von Klagsamkeit ist in unterschiedlichen Kulturen sehr unterschiedlich. Ob Müdigkeit infolge einer somatischen Erkrankung eine eigene Krankheit mit eigenem Diagnosecode ist oder nur ein Begleitsymptom unter vielen, ist Gegenstand der gesellschaftlichen Diskussion.

**Tab. 1:** Kriterien für die Diagnose Gesundheit und gesundes Leiden

1	Konnotation der verwendeten Begriffe und Symptombezeichnungen. Was ist mit der verwendeten Begrifflichkeit gemeint, wie ist sie zu operationalisieren?
2	Steht die vorgetragene Klage für ein (psycho)pathologisches Symptom?
3	Welche Zusatzsyndrome werden geklagt? Ergibt sich eine klinisch bekannte Syndromgestalt oder werden multiforme arbiträre Beschwerden vorgetragen?
4	Welche Intensität haben die Beschwerden? Liegt eine Teilhaberelevanz vor? Bagatellbeschwerden sind nicht krankheitswertig
5	Wie ist die Leidenstoleranz oder Resilienz der Person einzuschätzen, d. h. die Widerstandsfähigkeit gegenüber Anforderungen, Misslichkeiten oder Unwohlsein? Ein Mangel an Resilienz und eine erhöhte Tendenz zur Klagsamkeit ist für sich genommen keine Krankheit, sondern ein Persönlichkeitsstil. Ein faktenbasierter Beschwerdevortrag erfolgt in der Regel auf eher nüchterne und nicht auf aggravierende Art
6	Es ist das Persönlichkeitsmerkmal der Neigung zu einer verstärkten Selbstbeobachtung abzuklären, d. h. die Lage- bzw. Handlungsorientierung. Eine verstärkte Aufmerksamkeit auf die eigene Befindlichkeit führt zwingend zu einer verstärkten Wahrnehmung der aktuellen Befindlichkeiten, erhöhten Sensibilität und auch Selbstfürsorge, ohne dass dies ein krankhafter Prozess ist
7	Es ist der Grad der Verunsicherung durch erlebte Beschwerden zu explorieren wie auch die dadurch bedingten subjektiven Erklärungen, die wieder selbst Anlass zur Verunsicherung sein können. Die Informations- und Deutungsquellen sind zu erfragen, einschließlich ärztlicher Nocebo-Kommunikationen
8	Es sind die persönlichkeitsnahen Krankheitskonzepte bzw. dysfunktionalen Kognitionen zu explorieren, wie beispielsweise internale und externale Kontrollüberzeugungen oder die Tendenz zu biologistischen oder psychologistischen Welterklärungen
9	Der Kontext der Beschwerdenentstehung ist zu klären. Es gibt keinen Menschen ohne Anforderungen und Schwierigkeiten bei der Lebensbewältigung und damit assoziierten Beschwerden
10	Zu klären ist der Umgang mit den geklagten Beschwerden und eventuellen Einschränkungen, wie beispielsweise ein inadäquates Schonverhalten oder kämpferische Gegenreaktionen
11	Zu beurteilen ist die soziale Einbettung der Klage, die Funktionalität der Klage bzw. der Krankheitsgewinn
12	Es sind gesellschaftliche Konventionen und Normen zu berücksichtigen. Dies gilt auch unter einer transkulturellen Perspektive

Zusammengefasst zeigen die beschriebenen Validitätskriterien und diagnostischen Algorithmen, dass die Diagnose Gesundheit schwieriger zu stellen ist als die Feststellung einer Krankheit. Dies gilt grundsätzlich in der Medizin. Im Röntgenbild einen Tumor zu diagnostizieren ist z. B. vergleichsweise einfach, festzustellen, dass jemand keinen Tumor hat, jedoch aufwendig. Die Kriterien zeigen auch, dass eine Krankheit, z. B. eine zurückliegende Viruserkrankung, Tumor- oder Lebererkrankung, eine „gesunde Müdigkeit“ nicht ausschließt. In der S3-Leitlinie „Müdigkeit“ der AWMF wird dementsprechend auch warnend darauf hingewiesen, dass bei unspezifischen Befindensstörungen für Betroffene wie Ärzte die Versuchung groß ist, sich vorschnell auf unzureichend belegte Diagnosen und diagnostische Erklärungen zu einigen, weil Patienten sich dadurch häufig ernst genommen und entlastet fühlen, auch wenn solche Etikettierungen oft nicht evidenzbasiert sind und die Gefahr besteht, dass es dadurch zu einer eingeengten Perspektive kommen und für die Patienten gefährlich werden kann. Allerdings finden sich in der Leitlinie weder die Begriffe „gesund“ noch „Validierung“.

## Therapeutischer Umgang mit gesundem Leiden

Menschen, die unter unklaren Beschwerden wie Müdigkeit oder Burn-out leiden, tun zunächst einmal gut daran, Hilfe zu suchen. Wegen der vielfältigen differenzialdiagnostischen Möglichkeiten und wegen der Unsicherheit, ob nicht „etwas Ernstes“ zugrunde liegt, ist es legitim, eine professionelle Abklärung vorzunehmen. Dabei gibt es keine obligaten, sondern nur fallabhängige klinisch basierte Behandlungsansätze, die vorwiegend einen psychotherapeutischen Schwerpunkt haben [68–72].

Wie bereits beschrieben, kann eine Überdiagnostik zu Problemen führen. Es sollten nicht aus Unsicherheit weitreichende diagnostische Maßnahmen, Labor- und apparative Untersuchungen oder gar Therapiemaßnahmen eingeleitet werden, die das Problem verschärfen können. Es sollten nicht leichtfertig Krankheitsdiagnosen gestellt oder angedeutet werden, um eine Medikalisierung von Alltäglichkeiten zu vermeiden. Es ist untherapeutisch und kann als psychische Körperverletzung bezeichnet werden, wenn Patienten durch (Verdachts‑)Diagnosen, medizinische Überlegungen oder überfordernde Aufklärung verunsichert werden, bis hin zur Hypochondrisierung [62, 63, 73]. Es dürfen auch aus administrativen Gründen oder zum Zweck der Abrechnung keine Diagnosecodes vergeben werden, obwohl dies in der Praxis immer wieder zu beobachten ist.

Mit der Vergabe eines „M- oder Q‑Codes“ aus dem ICD kann diagnostisch deutlich gemacht werden, dass keine Krankheit vorliegt. Zugleich kann auch auf zugrunde liegende Lebensprobleme hingewiesen werden, seien es das Berufsleben mit Schichtarbeit, Familienprobleme oder sonstige Probleme in der Lebensführung. Die M‑ und Q‑Codes können damit bereits in die richtige diagnostische und vor allem therapeutische Richtung weisen.

Der erste „therapeutische“ Schritt ist die klar vermittelte Diagnose einer „gesunden Müdigkeit“. Der Betroffene braucht Sicherheit in der Einordnung des eigenen Zustands. Der zweite Schritt ist dann fast schon zwangsläufig die Frage, was man tun kann, um den eigenen Biorhythmus ernster zu nehmen. Dazu gehört, sich nicht gegen Müdigkeit zu wehren, sondern sie zu akzeptieren und anzunehmen, weil man biologisch nun einmal mit einem Biorhythmus geboren wurde. Dies gilt auch dann, wenn man faktisch nichts an der aktuellen Situation ändern kann, wie beispielsweise bei nächtlich schreienden Kindern.

In vielen Fällen sind die Probleme aber nicht von außen vorgegeben, sondern selbstgemacht. Die Mittagsmüdigkeit lässt sich durch einen Mittagsschlaf überwinden. Man muss ihn nur umsetzen und nicht meinen, dass man dadurch zu viel Zeit für angeblich Wichtigeres verliert. Die Bezeichnung „Nickerchen“ ist dabei deutlich Biorhythmus-freundlicher als „Powernapping“, das in der Presse als „Aufputscher“ oder „natürliches Doping“ angepriesen wird und sofort erkennen lässt, dass hier der Teufel mit dem Beelzebub ausgetrieben werden soll. Man könnte auch früher ins Bett gehen, weniger fernsehen oder sich einen an den eigenen Biorhythmus angelehnten regelmäßigen Tagesrhythmus zulegen. Aber die Umsetzung ist meist ebenso schwierig wie die Umsetzung anderer guter Vorsätze.

Abschließend ist im Umgang mit subjektiven Beschwerden auf 2 grundlegende Prinzipien in der Medizin hinzuweisen. Das erste ist „Präzisionsmedizin“ [74], worunter Präventions- und Therapiestrategien verstanden werden, die die Vielfalt der individuellen Eigenschaften berücksichtigen. Dies wird in letzter Zeit vermehrt mit Blick auf biologische Parameter diskutiert, ist aber seit jeher ein Kennzeichen der klinischen Medizin und speziell der Psychotherapie. Dabei sind unter einer ganzheitlichen biopsychosozialen Perspektive aber nicht nur biologische, sondern ebenso psychologische und soziale Parameter zu berücksichtigen, so wie oben in ihrer Vielfalt dargestellt und in der Internationalen Klassifikation der Funktionsfähigkeit, Behinderung und Gesundheit, ICF, vorgegeben [75]. Das zweite Prinzip ist „Watchful Waiting“ oder „aktives“ Nichtstun [76]. Auch wenn im konkreten Fall mit großer Sicherheit von einer Bagatellbeschwerde auszugehen ist, kann der weitere Verlauf durchaus etwas anderes zeigen.
